# Circadian clock-related genome-wide mendelian randomization identifies putatively genes for ulcerative colitis and its comorbidity

**DOI:** 10.1186/s12864-024-10003-z

**Published:** 2024-02-01

**Authors:** Mengfen Huang, Yuan Wu, Yiting Li, Xueru Chen, Jieni Feng, Zuming Li, Jiqiang Li, Jiankun Chen, Yue Lu, Yan Feng

**Affiliations:** 1 Guangzhou University of Chinese Medicine, Guangzhou, China; 2 The Second Clinical Medical College, Guangzhou University of Chinese Medicine, Guangzhou, China; 3 The First Clinical Medical College, Guangzhou University of Chinese Medicine, Guangzhou, China; 4 The Second Affiliated Hospital of Guangzhou University of Chinese Medicine (Guangdong Provincial Hospital of Chinese Medicine), Guangzhou, China

**Keywords:** Mendelian randomization, Circadian rhythm disorder, UC and its comorbidities, Pharmaceutical targets

## Abstract

**Background:**

Circadian rhythm is crucial to the function of the immune system. Disorders of the circadian rhythm can contribute to inflammatory diseases such as Ulcerative colitis (UC). This Mendelian Randomization (MR) analysis applies genetic tools to represent the aggregated statistical results of exposure to circadian rhythm disorders and UC and its comorbidities, allowing for causal inferences.

**Methods:**

Summary statistics of protein, DNA methylation and gene expression quantitative trait loci in individuals of European ancestry (pQTL, mQTL, and eQTL, respectively) were used. Genetic variants located within or near 152 circadian clock-related genes and closely related to circadian rhythm disorders were selected as instrumental variables. Causal relationships with UC and its comorbidities were then estimated through employed Summary data-based Mendelian Randomization (SMR) and Inverse-Variance-Weighted MR (IVW-MR).

**Results:**

Through preliminary SMR analysis, we identified a potential causal relationship between circadian clock-related genes and UC along with its comorbidities, which was further confirmed by IVW-MR analysis. Our study identified strong evidence of positive correlation involving seven overlapping genes (CSNK1E, OPRL1, PIWIL2, RORC, MAX, PPP5C, and AANAT) through MWAS and TWAS in UC, four overlapping genes (OPRL1, CHRNB2, FBXL17, and SIRT1) in UC with PSC, and three overlapping genes (ARNTL, USP7, and KRAS) in UC with arthropathy.

**Conclusions:**

This SMR study demonstrates the causal effect of circadian rhythm disorders in UC and its comorbidities. Furthermore, our investigation pinpointed candidate genes that could potentially serve as drug targets.

**Supplementary Information:**

The online version contains supplementary material available at 10.1186/s12864-024-10003-z.

## Key message

Circadian rhythm is crucial to the function of the immune system. Disorders of the circadian rhythm can lead to inflammatory diseases such as UC. MR, a technique utilizing genetic variation as instrumental variables, is employed to explore causal links between risk factors and disease outcomes. Our objective is to investigate the causal association between circadian rhythm disorders and UC along with its comorbidities, characterized by genetic susceptibility to circadian clock-related genes, through a comprehensive MR analysis.

**Supplementary Information:**

The online version contains supplementary material available at 10.1186/s12864-024-10003-z.

## Introduction

Ulcerative colitis (UC) is an immune-mediated chronic inflammatory bowel disease (IBD) characterized by persistent inflammation of the intestinal mucosa or lamina propria [1]. According to a widely accepted framework, the complexity of the environment and the host increases the susceptibility to UC, and events like disruption of the mucosal barrier, imbalance of the intestinal microbial flora, and abnormal stimulation of the intestinal immune response are what actually cause the disease [2]. Research reveals that circadian rhythm may be crucial in the pathogenesis of IBD, as sleep and melatonin may alter the immune system and intestinal microbiota, which may contribute to the incidence and progression of IBD [3, 4]. The circadian rhythm, also known as the circadian clock, is an endogenous “oscillator” in living things that controls nearly every aspect of biology and behavior, such as immune system function and metabolism, to ensure that internal physiology and the outside environment are in harmony [5, 6]. Previous studies have shown that the immune system’s dependence on circadian rhythm will influence the incidence and progression of IBD [7]. Circadian rhythm can control immune system function by controlling circulating lymphocytes, natural killer (NK) cells, antibody formation, complement level, cytokine synthesis, host-pathogen interaction, and induction of innate and adaptive immunity [4, 8, 9]. Additionally, chemokines and adhesion molecules on endothelial cells undergo diurnal variations, influencing the transport of leukocytes to intestinal tissue [10]. Consequently, the immune system depends on circadian rhythm, and a disruption of this cycle may result in inflammatory illnesses like IBD. Further, some studies have suggested that circadian rhythm disruption may be a risk factor contributing to the progress of severe IBD [11]. In order to better understand UC and its comorbidities, it is crucial to investigate the involvement of circadian rhythm disturbance.

Mendelian randomization (MR), which involves creating models using genetic variation as instrumental variables, is one technique used to investigate the causal link between risk factors and illness outcomes [12]. Using random distribution of genetic variants that alter exposure, MR is considered as a “natural” randomized controlled experiment [13]. It significantly removes the involvement of unobserved confounding variables and prevents reverse causality when compared to other study designs [14]. Genome-wide association studies (GWAS), which examine DNA sequence variations brought on by single nucleotide mutations, weed out single nucleotide polymorphisms (SNPs) that differ significantly from one other. These SNPs consistently link to the investigated risk variables. Numerous risk factors have been linked to an increasing number of genetic variants. To explore the association between these genetic variants and the intended outcome in GWAS, researchers can employ these genetic variations as instrumental variables, which represent the risk factors that are near to lifetime exposure [12]. Summary-data based MR (SMR) is an extension of the MR concept, primarily employed for analyzing associations among genotype, gene expression, and phenotype. SMR integrates and analyzes GWAS summary data with expression quantitative trait loci (eQTL) or DNA methylation quantitative trait loci (mQTL), developed to prioritize causal variation mediated by gene expression or DNA methylation [15]. Through the application of SMR, a study identified GPX1 as exhibiting a protective effect against UC, indicating its potential as a pharmacological target for these conditions [16].

The MR investigation looking for a possible causal link between circadian rhythm disruption and ulcerative colitis and its comorbidities was not discovered. Hence, in our study, our objective is to explore the causal association between circadian rhythm disorder and ulcerative colitis along with its comorbidities, characterized by genetic susceptibility of circadian clock-related genes through comprehensive MR analysis.

## Materials and methods

This study was carried out in accordance with the reporting guideline of the Strengthening the Reporting of Observational Studies in Epidemiology (STROBE, Supplementary Table 1) [17, 18].

### Study design

**Fig. 1:**
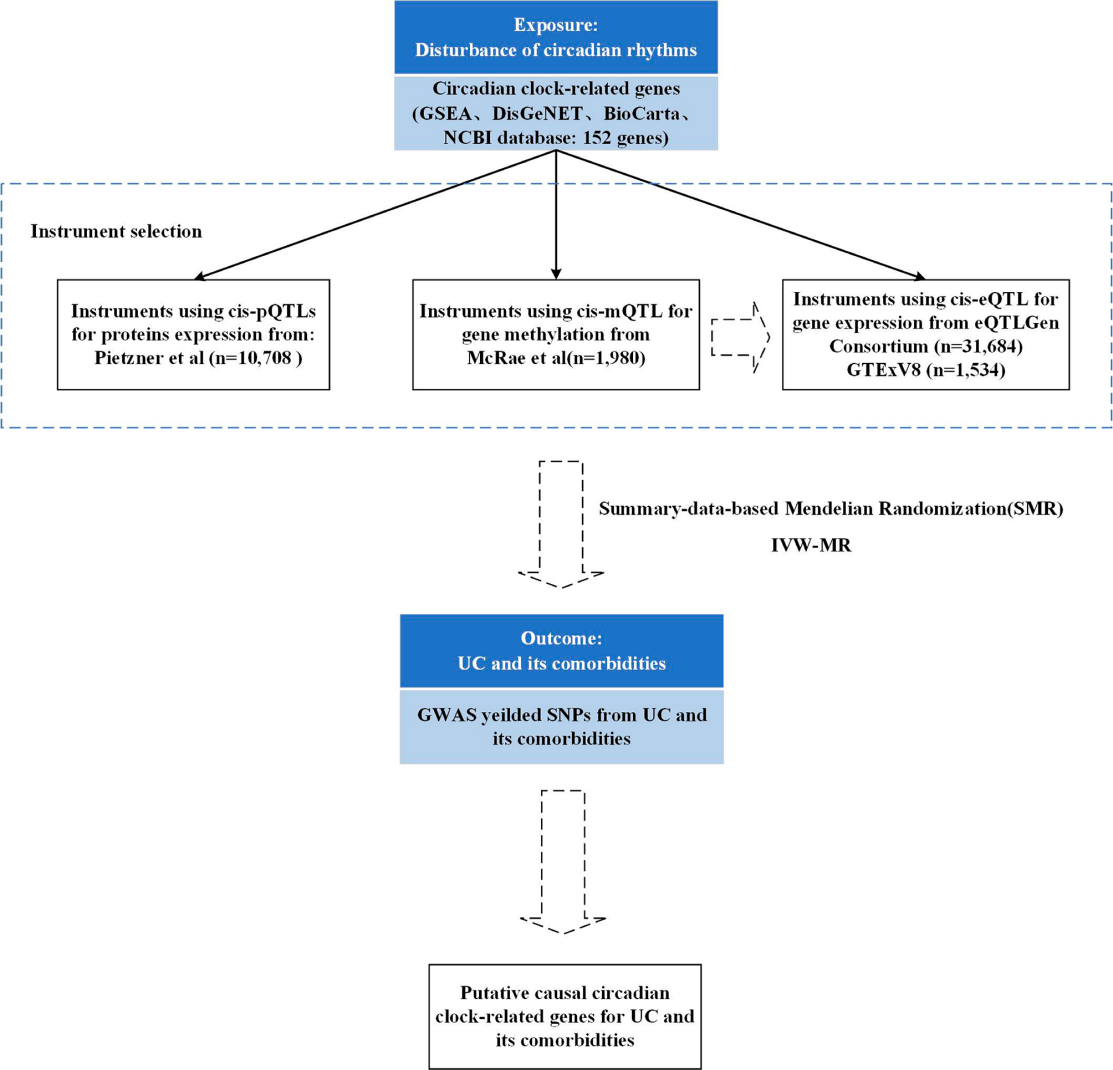
Summary of the study GSEA: Gene Set Enrichment Analysis, QTL: quantitative trait loci, IVW-MR: Inverse-Variance-Weighted MR, NCBI: National Center for Biotechnology Information, UC: Ulcerative colitis, GWAS: Genome-wide association studies, SNP: single nucleotide polymorphism

The quantitative trait loci (QTL) and GWAS studies that provided the summary-level data for this SMR analysis are both publicly available (Fig. 1). Participants in each of these investigations gave their informed permission, and each study was authorized by the appropriate institutional review boards.

### Patient and public involvement

It was not appropriate or possible to involve patients or the public in the design, or conduct, or reporting, or dissemination plans of our research.

### Data sources of circadian clock-related genes (CRGs)

CRGs were collected from the Gene Set Enrichment Analysis (GSEA) database (http://www.gsea-msigdb.org/gsea/msigdb), DisGeNET (https://www.disgenet.org), BioCarta (https://maayanlab.cloud/Harmonizome/dataset/Biocarta+Pathways), National Center for Biotechnology Information (NCBI) (https://www.ncbi.nlm.nih.gov) using “circadian clock” as the main search term, and 152 genes were obtained.

### Selection of genetic instruments

As shown in Table 1, we used available QTLs for CRGs. The expression QTL (eQTL) summary-level data were obtained from eQTLGen Consortium [19] (https://www.eqtlgen.org/) and GTEx Consortium V8 [20] (https://gtexportal.org/), relevant information is detailed in the Table 1. MR methylation QTL (mQTL) instruments for genetic variants stably correlated with circadian rhythm-related gene methylation were selected using blood mQTL summary data from a meta-analysis of two cohorts (*n* = 1980) [21]. We used the annotation file from Price et al. [22] to annotate the closest genes of DNA methylation probes. MR protein QTL (pQTL) instruments for genetic variants correlated with the expression of circadian rhythm-related protein were extracted using summary data from Pietzner et al. [23]. For eQTL, mQTL and pQTL data, only probes having at least one common (minor allele frequency [MAF] > 1%) cis-QTL passing the genome-wide significant threshold (P_QTL_<5 × 10^− 8^) [77], which associated with the expression of CRGs, were included in the SMR analyses. The cis region was defined as within 2 Mb of a probe in either direction.

To validate the observed association using eQTLs as instruments, we further devised an instrument by selecting SNPs within 100 kb windows from the target gene associated with the circadian clock at a genome-wide significance level (*p* < 5.0 × 10 ^− 8^) to serve as proxies for circadian clock-related exposure. To optimize the robustness of the instrument, SNPs utilized as instruments were deliberately chosen to exhibit low weak linkage disequilibrium (r^2^ < 0.30) with each other.

### Outcome sources

GWAS summary statistics for UC and its comorbidity were obtained from the FinnGen database, with a sample size of 341,859 for UC, 331,563 for UC with primary sclerosing cholangitis (PSC), and 339,549 for UC with arthropathy, respectively [24]. The study population was restricted to individuals with European ancestry.

**Table 1:** Information of eQTL and GWAS summary data

Characteristic	Resource	Sample size	Population ancestry
**eQTL data**			
Whole blood	eQTLGen Consortium	31,684	Predominantly European
	GTExV8	755	
Colon Sigmoid		373	
Colon Transverse		406	
**mQTL data**			
Whole blood	McRae et al.	1980	European
**pQTL data**			
Plasma	Pietzner et al.	10,708	European
**GWAS summary data**			
UC	FinnGen	ncases: 4460 ncontrols: 337,399	European
UC with PSC		ncases: 189 ncontrols: 331,374	
UC with arthropathy		ncases: 279 ncontrols: 339,270	

eQTL: expression quantitative trait loci, mQTL: methylation quantitative trait loci, pQTL: protein quantitative trait loci, UC: ulcerative colitis, PSC: primary sclerosing cholangitis, GTEx: Genotype-TissueExpression

### Statistical analyses

#### SMR and MR analysis

Adopting cis-QTLs as a tool, the summary-data based MR (SMR) approach was used to produce effect estimates. SMR analyzes the relationship between gene expression and an outcome of interest using summary-level data from GWAS and cis-QTL investigations [15, 78]. Allele harmonization and analysis were performed using SMR software (version 1.03, https://yanglab.westlake.edu.cn/software/smr/#Overview). MR analysis employed the inverse-variance weighted (IVW) approach, and we supplemented it with other approach, such as weighted-median estimator approach to enhance the robustness of our results [25, 26]. Odds ratio (OR) estimates of circadian rhythm dysregulation on the risk of UC and its comorbidity were calculated as follows: OR = exp(β), where OR is the odds ratio estimate per 1-ln increase in circadian clock genome levels and exp is the base of the natural logarithm.

### Sensitivity analysis

The SMR software was used to conduct the heterogeneity in the dependent instrument (HEIDI) test for the SMR technique to determine if the observed correlation between gene expression and the result was caused by a linkage scenario [15]. Genomes of European ancestry acquired from the 1000 Genomes Project Consortium were used as a reference for the linkage disequilibrium (LD) calculation [27]. The HEIDI test of *P* < 0.01 indicates that the association is probably due to linkage [28]. There might be horizontal pleiotropy if an SNP is linked to the expression of many genes. We found other neighboring genes within a 1 Mb window, whose expression was strongly correlated with the genetic instrumental mutation, and did SMR analysis to see if the expression of these genes was connected to the UC outcomes in order to evaluate the probability of horizontal pleiotropy. We carried out a series of sensitivity analyses to determine the robustness of the results of MR analysis, including using MR-Egger regression and Mendelian Randomization Pleiotropy Residual Sum and Outlier (MR-PRESSO) analysis, which detect horizontal pleiotropy. We calculated the Cochran’s Q value to assess heterogeneity of the SNPs used as instrument variants.

The Bonferroni adjustment was used to modify the threshold of significance level to account for multiple testing, thus strong evidence was suggested for *P* < 0.05/the number of probes, and suggestive evidence of 0.05/the number of probes ≤ *P* ≤ 0.05.

## Results

### Circadian clock genome-wide cis-QTLs

After SMR testing, 76 circadian clock-related transcripts from blood and 20 from the colon were obtained, respectively. To minimize the genome-wide type I error, P_eSMR_BLOOD_ <6.579 × 10^− 4^ (Bonferroni correction, *P* < 0.05/76) and P_eSMR_COLON_ <2.500 × 10^− 3^ (Bonferroni correction, *P* < 0.05/20) suggests strong evidence of an association, while 6.579 × 10^− 4^ < P_eSMR_BLOOD_ <0.05 or 2.500 × 10^− 3^ < P_eSMR_COLON_ < 0.05 indicating suggesting evidence.

For the causal effect between the DNA methylation of the circadian clock-related genome and outcomes, 3880 unique genetic loci were identified, and P_mSMR_<1.290 × 10^− 5^ (Bonferroni correction, *P* < 0.05/3880) suggests strong evidence of an association, while for the causal association between the circadian clock-related protein and outcomes, 19 circadian rhythm related protein expressions were selected, and P_pSMR_<2.631 × 10^− 3^ (Bonferroni correction, *P* < 0.05/19) suggests strong evidence of an association.

### SMR and MR analysis of UC

**Fig. 2:**
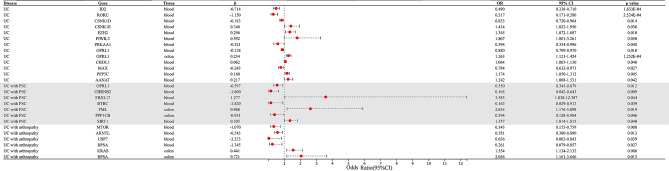
Summary-data-based Mendelian randomization (SMR) results for the association between the expression of circadian clock-related genes and risk of UC and it’s comorbiditiesSMR: summary-data-based Mendelian randomization, OR: odds ratio, UC: Ulcerative colitis, PSC: Primary sclerosing cholangitis

In SMR analysis of circadian clock genome-wide cis-eQTLs and UC (Fig. 2, Supplementary Table 2), results found strong evidence for the association of the increased expression of ID2 and RORC in the blood (equivalent to one standard deviation increase) with the lower risk of UC (OR 0.490, 95%CI 0.338–0.710; OR 0.317, 95%CI 0.171–0.586, respectively), while the increased expression of OPRL1 in colon correlated with a higher risk of UC (OR 1.263, 95%CI 1.121–1.424), indicating that upregulating the expression of ID2 and RORC in blood and downregulating the expression of OPRL1 in the colon might lower the risk of UC. Suggestive evidence was observed that the decreased expression of CSNK1D, PPKAA1, OPRL1, and MAX in blood were associated with lower risk of UC (OR 0.833, 95%CI 0.720–0.964; OR 0.594, 95%CI 0.354–0.996; OR 0.880, 95%CI 0.799–0.970; OR 0.784, 95%CI 0.672–0.973, respectively), while increased expression of CSNK1E, EZH2, PIWIL2, CHI3L1, PPP5C, and AANAT in blood were associated with a higher risk of UC (OR 1.414, 95%CI 1.022–1.956; OR 1.345, 95%CI 1.072–1.687; OR 1.807, 95%CI 1.001–3.261; OR 1.064, 95%CI 1.003–1.130; OR 1.174, 95%CI 1.050–1.312; OR 1.242, 95%CI 1.008–1.531, respectively).

**Fig. 3:**
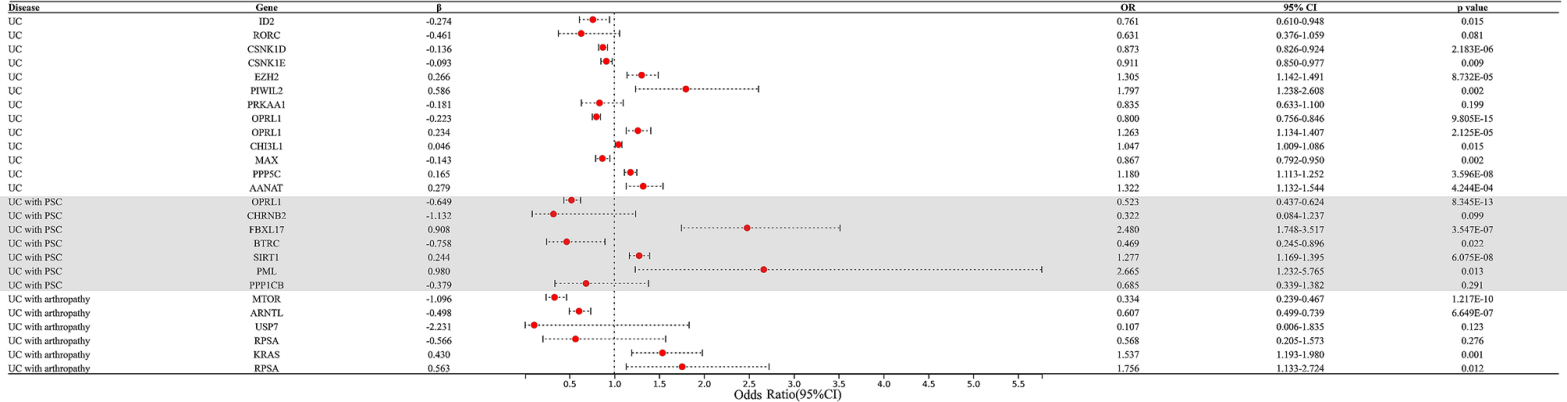
Mendelian randomization (MR) results for the association between the expression of circadian clock-related genes and risk of UC and it’s comorbidities. MR: Mendelian randomization, OR: odds ratio, UC: Ulcerative colitis, PSC: Primary sclerosing cholangitis

IVW-MR analysis also found evidence for the causal association between ID2, OPRL1 (both in colon and blood), CSNK1D, CSNK1E, MAX, EZH2, PIWIL2, CHI3L1, PPP5C, and AANAT with UC (OR 0.761, 95%CI 0.610–0.948; OR 1.263, 95%CI 1.134–1.407; OR 0.800, 95%CI 0.756–0.846; OR 0.873, 95%CI 0.826–0.924; OR 0.911, 95%CI 0.850–0.977; OR 0.867, 95%CI 0.792–0.950; OR 1.305, 95%CI 1.142–1.491; OR 1.800, 95%CI 1.238–2.608; OR 1.047, 95%CI 1.009–1.086; OR 1.180, 95%CI 1.113–1.252; OR 1.322, 95%CI 1.132–1.544, respectively) (Fig. 3, Supplementary Table 3). IVW-MR analysis further supporting a possible protective effect of ID2, CSNK1D, CSNK1E, and MAX against the development of UC, and suggesting EZH2, PIWIL2, CHI3L1, PPP5C, and AANAT as risk factors of the development of UC. In IVW-MR analysis, horizontal pleiotropy was observed in CSNK1E and MAX (*P* = 0.028; *P* = 0.013), while heterogeneity was identified in OPRL1.

### SMR and MR analysis of UC with PSC

In SMR analysis of circadian clock genome-wide cis-eQTLs and UC with PSC (Fig. 2, Supplementary Table 2), results found suggestive evidence for the association of the increased expression of CHRNB2, BTRC, and OPRL1 in blood with the lower risk of UC with PSC (OR 0.165, 95%CI 0.042–0.643; OR 0.162, 95%CI 0.029–0.912; OR 0.550, 95%CI 0.345–0.879, respectively), while increased expression of FBXL17 and SIRT1 correlated with a higher risk of UC with PSC (OR 3.585, 95%CI 1.038–12.387; OR 1.356, 95%CI 1.014–1.815, respectively). In the colon, it is suggestive that the increased expression of PML is correlated with a higher risk of UC with PSC (OR 2.634, 95%CI 1.176–5.898), while the increased expression of PPP1CB is correlated with a lower risk of UC with PSC (OR 0.394, 95%CI 0.158–0.984).

IVW-MR analysis also found evidence for the casual association between OPRL1, BTRC, FBXL17, SIRT1, and PML with UC along with PSC (OR 0.523, 95%CI 0.437–0.624; OR 0.469, 95%CI 0.245–0.896; OR 2.480, 95%CI 1.748–3.517; OR 1.277, 95%CI 1.169–1.395; OR 2.665, 95%CI 1.232–5.765, respectively) (Fig. 3, Supplementary Table 3). IVW-MR analysis provides additional support for a potential protective effect of OPRL1 and BTRC against the development of UC with PSC, while also suggesting FBXL17, SIRT1, and PML as potential risk factors. In IVW-MR analysis, horizontal pleiotropy was observed in FBXL17 and BTRC (*P* = 0.011; *P* = 0.013).

### SMR and MR analysis of UC with arthropathy

In SMR analysis of circadian clock genome-wide cis-eQTLs and UC with arthropathy (Fig. 2, Supplementary Table 2), results found suggestive evidence for the association of the increased expression of MTOR, ARNTL, USP7, and RPSA in blood with the lower risk of UC with arthropathy (OR 0.343, 95%CI 0.155–0.759; OR 0.581, 95%CI 0.380–0.890; OR 0.036, 95%CI 0.002–0.843; OR 0.261, 95%CI 0.079–0.857, respectively). In the colon, it is suggestive that the increased expression of KRAS and RPSA are correlated with a higher risk of UC with arthropathy (OR 1.555, 95%CI 1.134–2.132; OR 2.057, 95%CI 1.161–3.646, respectively).

IVW-MR analysis also found evidence for the casual association between MTOR, ARNTL, KRAS, and RPSA with UC along with arthropathy ((OR 0.334, 95%CI 0.239–0.467; OR 0.607, 95%CI 0.499–0.739; OR 1.537, 95%CI 1.927–1.980; OR 1.756, 95%CI 1.133–2.724, respectively) (Fig. 3, Supplementary Table 3).

IVW-MR analysis provides added support for a potential protective effect of MTOR and ARNTL against the development of UC with arthropathy, while also indicating KRAS and RPSA as potential risk factors.

### SMR analysis of circadian clock genome-wide cis-pQTLs and outcomes

Only 19 hypothesized circadian rhythm-related SNPs were retrieved from cis-pQTLs, and based on our recommended threshold following the SMR analysis, no causal connection was discovered. One explanation could be that the pQTL datasets are not fully established and only a small number of genetic variants that are strongly related with protein levels were found.

### SMR analysis of circadian clock genome-wide cis-mQTLs and outcomes

To determine if causal genes revealed by TWAS show a similar correlation at the methylome level, we performed methylome-wide association studies (MWAS) to provide insight into gene expression. In SMR analysis of circadian clock genome-wide cis-mQTLs and outcomes (Supplementary Fig. 2, Supplementary Table 4), results found a total of 38 association signals across 32 unique genetic loci for UC, 37 association signals across 30 unique genetic loci for UC with PSC, and 29 association signals across 26 unique genetic loci for UC with arthropathy. Seven overlapping genes (CSNK1E, OPRL1, PIWIL2, RORC, MAX, PPP5C, and AANAT) between MWAS and TWAS were identified in UC, four overlapping genes (OPRL1, CHRNB2, FBXL17, and SIRT1) in UC with PSC, and three overlapping genes (ARNTL, USP7, and KRAS) in UC with arthropathy.

## Discussion

The circadian rhythm coordinates daily physiological and behavioral cycles, enabling organisms to synchronize with the time of day. The circadian rhythm is also found in intestinal epithelial cells, where it plays a critical role in maintaining homeostasis, cell division, and permeability [29]. In our study, we indicated that circadian rhythm dysregulation characterized by genetic predisposition has a causal association on UC and its comorbidities, and identified important putative causal CRGs as follows: (1) CSNK1E, OPRL1, PIWIL2, RORC, MAX, PPP5C, and AANAT for UC; (2) OPRL1, CHRNB2, FBXL17, and SIRT1 for UC with PSC; (3) ARNTL, USP7, and KRAS for UC with arthropathy. Our findings demonstrate that genetic factors influencing circadian rhythm dysregulation were related to the risk of UC and its comorbidities in a comorbidity type-specific way, providing strong support for the underlying mechanisms relating genetic loci, gene expression, and methylation to UC and its comorbidities.

Clock gene family member CSNK1E controls signaling pathways connected to the circadian molecular clock [30]. It has been studied for decades how CSNK1E functions physiologically and it was found to be associated with cognitive deficits in schizophrenia [31], survival of several cancer [32, 33], heroin addiction, and opioid sensitivity [34]. Studies have shown that CSNK1E was upregulated in UC patients [35]. According to the DRUGBANK database (https://go.drugbank.com/), Seliciclib which targets CSNK1E has been investigating the mechanisms of chronic inflammation disorders. OPRL1 encodes a G protein-coupled receptor for nociceptin, an endogenous opioid-related neuropeptide, which is crucial in pain perception and nociception [36]. A previous study has shown that the neuropeptide nociceptin/orphanin’ FQ (N/OFQ) has been identified as a promising immunomodulator for inflammatory bowel diseases [37]. RORC, the core genes of the circadian clock, is a critical transcription factor for Th17 polarization and function, and it plays a significant role in autoimmunity and inflammation [38, 39]. It is proved that RORC mRNA expression was increased in patients with UC [40, 79]. PIWIL2 is a member of the gene subfamily known as P-element-induced wimpy testis/Argonaute, whose members are distinguished by conversed PAZ and PIWI domains. These genes are the first class of genes recognized to be necessary for stem cell self-renewal in a variety of animals [41, 42]. MAX, the MYC-associated factor X, plays a regulatory role in clock gene expression, contributing to maintaining a balance between the positive and negative elements of the molecular clock machinery [43]. MAX is a gene implicated in the expression of glycolytic enzymes, influencing breast cancer cell proliferation and migration, as well as anaplastic large cell lymphoma. MAX inactivation represents an early event in the development of gastrointestinal stromal tumors [44–46]. PPP5C, a member of the Protein Phosphatase 5 (PP5) family, functions as a serine/threonine protein phosphatase and has been identified as a regulator of the mammalian circadian clock [47]. PPP5C plays a crucial regulatory role in multiple signaling pathways through its interactions with various transcription factors and proteins. Dysregulation or abnormal activity of PPP5C is implicated in various diseases, including several cancers, obesity, and Alzheimer’s disease [48–52]. Arylalkylamine N-acetyltransferase (AANAT) is a pivotal enzyme in the biosynthesis of melatonin, a crucial regulator of the circadian rhythm, playing a significant role in regulating the circadian clock and influencing various physiological processes, including sleep-wake cycles and seasonal behaviors [53]. Research has revealed elevated levels of AANAT in the colonic mucosa of patients with UC, potentially indicating melatonin synthesis may related with the development of UC [54]. However, its causal effect with UC is not clear. In this study, we demonstrated that gene expression and methylation of CSNK1E, OPRL1, PIWIL2, RORC, MAX, PPP5C, and AANAT have a causal relationship with UC.

CHRNB2 is a crucial component of the nicotinic acetylcholine receptor and is connected to nicotine dependence, epilepsy, and cancer patient metastasis [55, 56].The gene FBXL17 encodes a little-studied member of the F-box family of proteins, which are essential for the ubiquitin conjugation pathway and control important cellular functions like cell cycle progression, cell signaling, and receptor recycling. These processes all require quick alteration in protein levels [57]. SIRT1, a nucleus-localized member of the sirtuin family, crucially regulates the circadian clock by deacetylating key components like CLOCK and BMAL1 [58]. Dysregulation of SIRT1 can impact mitochondrial function, metabolic homeostasis, and circadian synchronization [59]. As a therapeutic target, SIRT1 holds promise for conditions such as cardiovascular diseases, cancer, metabolic disorders, and inflammation [60, 80]. Previous studies have revealed that SIRT1, through mechanisms involving alterations in the intestinal microbiota and suppression of the NF-κB pathway, plays a protective role in intestinal inflammation [61–64]. Additionally, SIRT1 contributes to heightened intestinal barrier integrity and reduced apoptosis of intestinal epithelial cells by suppressing ER stress-mediated apoptotic proteins like CHOP and caspase-12, thereby alleviating UC [65]. However, there is little evidence available concerning their role in UC or PSC. Our results showed a causal relationship between the gene expression and methylation of OPRL1, CHRNB2, FBXL17, and SIRT1 and UC with PSC.

ARNTL, also known as BMAL1, serves as a core and master regulator of the circadian clock [66]. It has been demonstrated that ARNTL controls hepatic drug metabolism, resulting in dosing time-dependent pharmacokinetics and pharmacological action. ARNTL participates in metabolic homeostasis including lipid, glucose, and cholesterol [67]. ARNTL is essential for treating colitis, as demonstrated by a prior study that found the ARNTL-knockout mice model caused more severe colitis [68]. Additionally, ARNTL is crucial for osteoarthritis chondrocyte growth and cartilage tissue integrity [69–71]. Deubiquitinating enzyme USP7 has a role in the development of IBD by controlling Foxp3 expression and thereby impairing Treg cell functioning [72, 73]. Moreover, in rheumatoid arthritis, USP7 is connected to the migration of synoviocytes that resemble fibroblasts [74]. KRAS plays a pivotal role in transmitting signals from cell surface receptors to the nucleus, and dysregulation or mutations in the KRAS gene are commonly linked to various cancers, particularly colorectal cancer, underscoring its significance as a target for cancer research and therapeutic interventions [75, 81]. Studies have indicated that the activity of the RAS/MAPK pathway modulates the circadian period and, potentially by influencing the transcriptional activity of CLOCK/BMAL1 [76]. Our results were consistent in that there is a causal relationship between the gene expression and methylation of ARNTL, USP7, KRAS and UC with arthropathy, and can be the potential target of UC with arthropathy.

The primary strength of the current work lies in the utilization of multi-omics MR to investigate the association between circadian rhythm dysregulation, as determined by genetic susceptibility to known CRGs, and UC along with comorbidities. Second, we limited our study samples to individuals of European ancestry, thereby reducing biases stemming from diverse genetic origins.

Several restrictions apply to this study as well. Although we drew from a variety of large GWAS data sources, neither the available eQTL dataset nor the mQTL dataset contained information on genetic variants related to gene expression or methylation levels in the X chromosome, Y chromosome. Moreover, limited genetic variants representing the expression of the circadian rhythm protein were found. Second, based on the software resources now available, we are unable to determine the direction of the association using bi-directional MR since the GWAS dataset that directly indicated circadian rhythm disruption is not accessible. Our study recognizes the potential limitation of horizontal pleiotropy, adding complexity to causal inference. Despite employing MR-Egger regression to address this concern, caution is warranted in interpreting results due to method assumptions and sensitivity.

This study highlights the significance of circadian rhythm dysregulation in the pathogenesis of UC and its comorbidities by using MR to evaluate the potential causal association between circadian rhythm dysregulation, which is characterized by a genetic predisposition in CRGs, and UC and its comorbidities. In-depth study of the underlying biological pathways might be done using the discovered candidate genes as possible pharmaceutical targets for UC prevention and therapy.

### Electronic supplementary material

Below is the link to the electronic supplementary material.

Supplementary Material 1

Supplementary Material 2

## Data Availability

The peer-reviewed articles indicated in table 1 and cited in this study provide the data that were used in this analysis. Summary statistics for GWAS are publicly assessable for download. Requests can be made for the statistical code required to duplicate the findings in the paper.

## Abbreviations

CRGs: Circadian Clock-related Genes
eQTL: expression QTL
GSEA: Gene Set Enrichment Analysis
GWAS: Genome-wide Association Studies
HEIDI: Heterogeneity in the Dependent Instrument
IBD: Inflammatory Bowel Disease
IVW-MR: Inverse-Variance-Weighted MR
LD: Linkage Disequilibrium
mQTL: methylation QTL
MR: Mendelian Randomization
MWAS: Methylome-wide Association Studies
NCBI: National Center for Biotechnology Information
NK: Natural Killer
OR: Odds Ratio
pQTL: protein QTL
PSC: Primary Sclerosing Cholangitis
QTL: Quantitative Trait Loci
SMR: Summary data-based MR
SNPs: Single Nucleotide Polymorphisms
UC: Ulcerative Colitis
